# Women’s preferences and mode of delivery in public and private hospitals: a prospective cohort study

**DOI:** 10.1186/s12884-016-0824-0

**Published:** 2016-02-08

**Authors:** Agustina Mazzoni, Fernando Althabe, Laura Gutierrez, Luz Gibbons, Nancy H. Liu, Ana María Bonotti, Gustavo H. Izbizky, Marta Ferrary, Nora Viergue, Silvia I. Vigil, Gabriela Zalazar Denett, José M. Belizán

**Affiliations:** Mother and Child’s Health Research Department, Institute for Clinical Effectiveness and Health Policy (IECS), Dr. Emilio Ravignani 2024, C1414CPV Buenos Aires, Argentina; Institute for Clinical Effectiveness and Health Policy (IECS), Dr. Emilio Ravignani 2024, C1414CPV Buenos Aires, Argentina; UCSF General Internal Medicine, 3333 California Street, Box 0856, San Francisco, CA 94118 USA; Maternal and Child Health Program, Ministry of Health, 483 1/2 7th Avenue, 1900 La Plata, Buenos Aires Province Argentina; Department of Obstetrics, Hospital Italiano de Buenos Aires, Juan D. Perón 4190, C1181ACH Buenos Aires, Buenos Aires Argentina; Obstetrics Unit, Hospital Magdalena V. de Martínez, Avenida Constituyentes 395, General Pacheco, Tigre, 1617 Buenos Aires Province Argentina; Centro de Educación Médica e Investigaciones Clínicas “Norberto Quirno” (CEMIC), Hospital Universitario Saavedra, Av. Galván 4102, 1431FWO Buenos Aires, Argentina; Obstetrics Unit, Hospital Británico de Buenos Aires, Perdriel 74, C1280AEB Buenos Aires, Argentina; Obstetrics Unit, Hospital Materno Infantil Dr. Carlos Gianantonio, Diego Palma 505, San Isidro, Buenos Aires Province Argentina

**Keywords:** Caesarean section, Childbirth, Choice, Obstetric, Preference

## Abstract

**Background:**

Rates of caesarean section have steadily increased in most middle- and high-income countries over the last few decades without medical justification. Maternal request is one of the frequently cited non-medical factors contributing to this trend. The objectives of this study were to assess pregnant women’s preferences regarding mode of delivery and to compare actual caesarean section rates in the public and private sectors.

**Methods:**

A prospective cohort study was conducted in two public and three private hospitals in Buenos Aires, Argentina. 382 nulliparous pregnant women (183 from the private sector and 199 from the public sector) aged 18 to 35 years, with single pregnancies over 32 weeks of gestational age were enrolled during antenatal care visits between October 2010 and September 2011. We excluded women with pregnancies resulting from assisted fertility, women with known pre-existing major diseases or, with pregnancy complications, or with a medical indication of elective cesarean section. We used two different approaches to assess women’s preferences: a survey using a tailored questionnaire, and a discrete choice experiment.

**Results:**

Only 8 and 6 % of the healthy nulliparous women in the public and private sectors, respectively, expressed a preference for caesarean section. Fear of pain and safety were the most frequently expressed reasons for preferring caesarean section. When reasons for delivery mode were assessed by a discrete choice experiment, women placed the most emphasis on sex after childbirth. Of women who expressed their preference for vaginal delivery, 34 and 40 % ended their pregnancies by caesarean section in public and private hospitals, respectively.

**Conclusions:**

The preference for caesarean section is low among healthy nulliparous women in Buenos Aires. The reasons why these women had a rate of more than 35 % caesarean sections are unlikely related to their preferences for mode of delivery.

**Electronic supplementary material:**

The online version of this article (doi:10.1186/s12884-016-0824-0) contains supplementary material, which is available to authorized users.

## Background

Rates of cesarean section (CS) have steadily increased in most middle- and high-income countries over the last few decades without medical justification [1–3]. Maternal request is one of the frequently cited non-medical factors contributing to this trend [4–6]. However, studies show that few women actually prefer CS [7–12]. In a recent systematic review and meta-analysis we found that only 16 % of women in a wide variety of countries expressed a preference for CS [13]. Several factors were associated with a higher preference for CS, such as having had a prior CS and living in a middle-income country.

Thus, although individual demand for CS has been suggested as a factor related to increasing CS rates [6, 14], there might be other important non-medical factors that have not been identified. The different CS rates at public and private maternity hospitals suggest that either differences in patient preferences for mode of delivery, or the different organization of prenatal and delivery care between these two settings could influence delivery outcomes [5, 9]. However, it is not clear whether delivery preferences differ across these two settings and there is no body of evidence on this topic [13]. Potter and colleagues surveyed pregnant women attending public and private institutions in Brazil [9], and found that preferences were similar in both sectors, with more than 80 % of women in favor of vaginal delivery (VD). More recently, our group has examined various motivational factors related to women’s preferences for mode of delivery among pregnant women attending public and private hospitals. For most participants, vaginal delivery was viewed as a normal, healthy, and a natural mode of delivery, except in the case of a medical indication for CS, which was viewed as a medical decision [15].

The objectives of this study were to examine the preferences for mode of delivery for pregnant women delivering in public and private hospitals in Buenos Aires Province, Argentina, and to compare the CS rates for women who expressed a preference for vaginal delivery across public and private hospitals. Additionally, we examined the reasons related to women’s preferences.

## Methods

This was a prospective, cohort study. Enrolled women were asked their preference for delivery type during the third trimester. Women were followed-up until delivery when their actual mode of delivery was assessed.

The primary objectives were twofold: 1) to assess women’s preferences regarding mode of delivery in the third trimester of pregnancy, and 2) to compare actual CS rates in women in public versus private sectors who expressed a preference for vaginal delivery. The secondary objectives included the following: 1) to compare the preferences for mode of delivery between public and private sector women, and 2) to describe the major reasons stated for the women’s preferences.

The study was conducted in two public hospitals and three private hospitals in and around the City of Buenos Aires attending more than 2000 deliveries per year. The hospitals were chosen based on a convenience sample of locations where other research studies have been conducted by our research organizations.

In Argentina, 99 % of deliveries occur at hospitals [16] and the health care system is composed of three sectors: a public sector, a social security sector, and a private sector. The public sector is financed by the Ministry of Health and its main beneficiaries are persons without health insurance, usually from lower socioeconomic groups. The social security sector is grounded in the principle of social insurance, which requires all employers and employees to make payments to a trust fund. This sector provides services for a variety of institutions, which vary greatly depending on their employee base and the medical insurance coverage provided. The private sector provides service to individuals of high socioeconomic status who may have different types of pre-paid health insurance packages. According to national data, 49 % of the women who delivered in 2013 was covered by social security or private insurance, and 42 % by public health system [16]. For the purposes of this study, the public sector is defined as individuals from the public and social security sectors.

### Participants

Between October 2010 and September 2011, we enrolled nulliparous pregnant women, aged 18 to 35 years, with singleton pregnancies and a live fetus over 32 weeks of gestational age. These women attended prenatal care at the participating hospitals, and planned to deliver at the same hospital. We excluded women with fertility-assisted pregnancies, known pre-existing major diseases, with pregnancy complications, or with a medical indication of elective CS.

We restricted the sample to women between the ages of 18 and 35 years old, in order to maintain a similar age distribution between the private and public hospitals and to reduce the potential confounding due to differences in the age of participants across hospital types. Age is a major determinant of women’s preferences for mode of delivery [17], particularly in the nulliparous subgroup. In Argentina, the age distribution of pregnant women between the private and public sectors varies dramatically; the proportion of pregnant women over 35 years old is higher in the private sector. Conversely, public hospitals have a higher proportion of pregnant adolescents than private hospitals [18, 19].

### Procedures

We used two different approaches to assess women’s preferences: 1) a survey using a tailored questionnaire, and 2) a discrete choice experiment.

For the survey, we adapted a questionnaire based on our previous research study evaluating the hypothesis that a hospital policy of mandatory second opinion, based on the best existing scientific evidence, reduces the hospital cesarean section rate [20]. This survey was further tailored based on the results of a formative research conducted to understand women’ s preferences and motivational factors for mode of delivery [15], and on the results of a systematic review or literature on women’s preferences for cesarean section [13]. Trained interviewers conducted the interviews and collected follow-up information from participating women during prenatal care visits in a designated quiet room in the prenatal care area of both public and private hospitals. Women who agreed to participate provided written informed consent. Contact information was collected for the follow-up at delivery. We enrolled all consecutive eligible women attending prenatal care during a pre-defined recruitment period lasting 6 months.

Additionally, we conducted a discrete choice experiment (DCE) to evaluate women’s preferences about mode of delivery. DCE is a quantitative method for eliciting preferences, which allows estimation of the relative importance of different characteristics or attributes when considered simultaneously towards one decision or another [21]. Because mode of delivery is a multi-faceted health decision composed of various advantages and disadvantages (for example, time in hospital, presence of a scar, pain) a DCE is an appropriate and effective approach to measure factors related to each women’s preference.

The discrete-choice experiment simultaneously considers different aspects involved in women’s preference for certain health decisions. Women choose between different scenarios, making trade- offs between different attributes or characteristics (for example, episiotomy, pain, recovery), as they cannot choose the best levels of all attributes [22]. It has been used in health research including evaluations of the maternal health care process and the assessment of preferences for intrapartum care [22–25].

We included five attributes that were found to be important determinants of women’s choice of delivery preference in our formative research [15] and through a literature review [7, 10–14, 26–28]. The attributes and their levels were: a) Possibility of scheduling the date of delivery (Yes/No), b) Episiotomy (Yes/No), c) Sexual function at 6 months post partum (the same than before delivery/worse than before delivery) d) Pain during delivery (mild/moderate/severe) e) Recovery after delivery (less than 1 week/between 1 and 2 weeks/more than 2 weeks). We sequentially presented to women 14 choice sets in separate cards. Each choice set was composed of two profiles; each profile described a combination of different levels of the 5 selected attributes. An example of one choice set is shown in Additional file 1. We asked women to choose which profile they preferred. We assessed rationality using one choice set where one profile was better than the other on all attributes (the “ideal profile”). Using this choice set allowed us to assess whether women were making rational choices. Additionally, with the objective of evaluating consistency of women’s responses, one choice set was presented twice in the sequence of choice sets presented to them.

Both the questionnaire and the DCE were piloted to evaluate the readability, comprehension, relevance and length of time to complete the assessment. The pilot study was conducted in 60 women, 30 each from the private and public sectors, who were receiving prenatal care at institutions similar to those selected for the study (data not shown).

To obtain data regarding the actual mode of delivery, interviewers checked the delivery logbook on a daily basis to determine when each participant delivered her baby. When the woman was identified, data were extracted from her clinical record, including mode of delivery, gestational age at delivery, CS indication (if applicable), type of initiation of labor (spontaneous or induced), and neonatal data. In the event that the delivery occurred in a non-participating hospital, information on the mode of delivery was requested from the woman using the contact information secured during her enrolment in the study.

### Statistical analysis

We conducted a descriptive analysis of the women’s sociodemographics characteristics separately in the public and private sectors. We report the proportion of women who prefer a vaginal and cesarean section by health care sector. According to the preference for mode of delivery, we report the reasons for this preference and the level of agreement to a set of closed-ended statements about preference for each delivery type. We also present the proportion of actual elective and intra-partum CS by preference type and health care sector. Chi-square tests were used to compare proportions.

Assuming an estimated preference of cesarean section of 15 % in each sector and a precision of 5 %, a sample size of 400 women (200 women per sector, public and private) was needed. This sample size estimation was enough to compare the CS rates in women preferring vaginal deliveries between private and public hospitals, assuming a CS rate of 25 % in the public sector versus 50 % in the private sector.

For the DCE analysis, twelve choice sets were created in SPSS based on an orthogonal design. We used a conditional logit model to analyze the DCE. Preference for mode of delivery was the dependent variable (i.e., a binary variable representing the woman’s choice). The five different attributes were the independent variables. We accounted for the potential correlation in responses between the fourteen choices completed by each woman and estimated a model for the private and public sectors separately.

We used a special procedure in SAS 9.1.3 called multinomial discrete choice [21]. For each model we report the beta coefficient with the 95 % confidence interval. The larger the coefficient associated with a given attribute, the greater the impact of a unit change in that attribute on respondents’ overall utility.

### Ethical approvals

The protocol and the informed consent documents were approved by the Ethics Committees of all participating hospitals between December 2008 and July 2011: Comité de Ética en Investigación de CEMIC; Comité de Ética de Protocolos de Investigación del Hospital Italiano de Buenos Aires; Comité de Revisión Institucional del Hospital Británico de Buenos Aires; Comité de Docencia e Investigación/Comité de Bioética del Hospital M.V. de Martínez; and Comité de Ética del Hospital Materno-Infantil de San Isidro Dr. Carlos Gianantonio. The documents were also approved by the Comité de Ética Central del Ministerio de Salud de la Provincia de Buenos Aires, Argentina (Central Ethics Committee of the Ministry of Health of the Province of Buenos Aires, Argentina) [29] on August 2010; and Tulane Human Research Protection Program, Institutional Review Boards, Tulane University, USA [30], on April 2010.

## Results

We enrolled 382 women, 183 from the private sector and 199 from the public sector. Only one woman was lost to follow-up. Table 1 reports the sociodemographic characteristics of women recruited from private and public health care hospitals. Public sector women were younger, had less formal education, less likely to be in a stable relationship, and were less employed. A larger percentage of women in the public sector were foreign-born and a smaller percentage of these women were employed.

**Table 1:** Women’s socio-demographic characteristics

	Public sector (*N* = 199)	Private sector (*N* = 183)
	n (%)	n (%)
Age (years)		
≤19	62 (31.2 %)	2 (1.1 %)
20–29	120 (60.3 %)	78 (42.6 %)
≥30	17 (8.5 %)	103 (56.3 %)
Education		
Elementary school	35 (17.7 %)	0 (0 %)
High school	142 (71.7 %)	32 (17.5 %)
Tertiary/University	21 (10.6 %)	151 (82.5 %)
Nationality		
Argentinean	165 (83.3 %)	178 (97.3 %)
Other	33 (16.7 %)	5 (2.7 %)
Marital status		
Married or stable partner	138 (69.7 %)	171 (93.4 %)
No partner	60 (30.3 %)	12 (6.6 %)
Work status		
Employed	31 (15.6 %)	150 (83.8 %)
Health-related work^a^	0 (0 %)	26 (14.5 %)
Unemployed	168 (84.4 %)	29 (16.2 %)

^a^Health-related work: any employment in the health sector

Despite these different characteristics, we observed no difference between women’s preferences for mode of delivery. Only 8 % (*n* = 16) of women in the public sector, and 6 % (*n* = 11) of women in the private sector expressed a preference for cesarean section (*p* = 0.55). 7 % (*n* = 12) of women in the private sector and none in the public expressed to have “no preference” for mode of delivery.

**Fig. 1:**
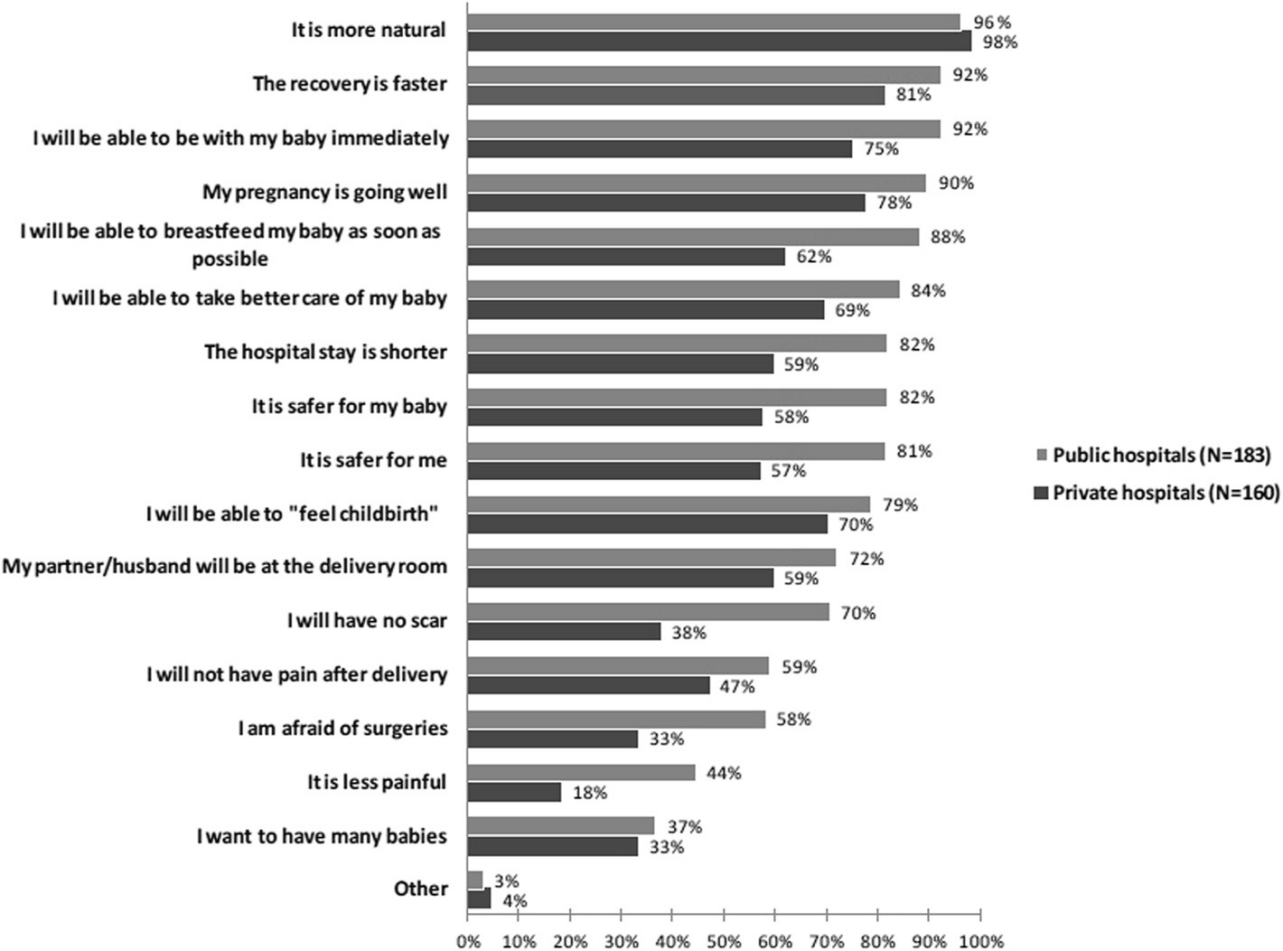
Women’s answers to the closed-ended question: “Why do you prefer to have the baby by vaginal delivery?” Percentage of women who "strongly agree" and "agree" by heath care sector (in descending order of responses in the public sector)

We asked women to provide reasons for their preference using an open-ended format. Among women who preferred vaginal delivery, the most frequent reasons provided were that vaginal delivery was viewed as a more “natural” way of giving birth (34.7 and 44.8 % of women in the public and private sectors, respectively), and that it facilitated an easier recovery (21.1 and 21.9 % in the public and private sectors, respectively). Fear/avoidance of surgeries was another common response, expressed by 12.6 and 13.7 % of women in the public and private sectors, respectively (Table 2). When women were asked their level of agreement with a pre-specified list of potential reasons for preferring vaginal delivery, the highest rated reasons (among “strongly agree” and “agree”, combined) in public and private sectors, respectively, were for: “It is more natural” (96 and 98 %), and “The recovery is faster” (92 and 81 %) (Fig. 1). Women from the public sector reported higher levels of agreement for all responses compared to women in the private sector.

**Table 2:** Women’s answers to the open-ended question: " Why do you prefer to have the baby by vaginal delivery?"

	Public sector (*N* = 183)	Private sector (*N* = 160)
	n (%)	n (%)
It is natural	65 (34.7 %)	82 (44.8 %)
The recovery is faster/better	42 (21.1 %)	40 (21.9 %)
Fear/avoidance of surgery	25 (12.6 %)	25 (13.7 %)
Feel childbirth/Experience delivery	30 (15.1 %)	19 (10.4 %)
Less painful	18 (9.0 %)	2 (1.1 %)
Safer	8 (4.0 %)	4 (2.2 %)
Better care of the baby after delivery	6 (3.0 %)	4 (2.2 %)
No scar	8 (4.0 %)	0 (0.0 %)
It is nicer	3 (1.5 %)	0 (0.0 %)
Shorter hospital admission	3 (1.5 %)	0 (0.0 %)
Don’t know	0 (0.0 %)	3 (1.6 %)
Other reason	33 (18.0 %)	26 (16.2 %)

Categories are not mutually exclusive

Among women who expressed a preference for cesarean section, 10 out of 16 women in the public sector and 4 out of 11 women in the private sector cited fear of the pain associated with vaginal delivery as the main reason. Other responses included: “fear”, “possibility to schedule the date”, “more comfortable”, “safer”, and “avoidance of episiotomy”. Responses for women in the public and private sectors varied when we asked participants their level of agreement with a pre-specified list of potential reasons for preferring CS. The highest rates of “strongly agree” and “agree” in the public sector were for “Safer for me” (12/16), “It is less painful” (11/16) and “To avoid episiotomy or vaginal tears” (11/16). In the private sector, the most frequently selected reasons were “To avoid episiotomy or vaginal tears” (8/11), “It is less painful” (7/11), and “It is faster than vaginal delivery” (6/11) and “The baby does not suffer” (6/11).

### Results of the discrete-choice experiment

Table 3 shows the results of the discrete choice model for each sector. In the public sector, four out of five attributes were significant predictors of the women’s choice. The direction of the statistically significant regression coefficients in the models indicate that, in order of strength, pregnant women in our sample prefer to: have the same sexual function as before delivery, have a faster recovery after delivery, have a less painful experience, and avoid episiotomy. The possibility of scheduling the delivery was not a significant factor for women in the public sector (*p* = 0.6314). In the private sector, each of the five attributes were significant predictors of the choice of women. In order of strength, women prefer to: have the same sexual function as before delivery, have a less painful experience, avoid episiotomy, have a faster recovery after delivery, and be able to schedule the delivery. For both public and private sectors, sex after childbirth was the attribute that women gave the most importance.

**Table 3:** Results of the regression model of the discrete choice experiment

Attributes	Public sector			Private sector		
	Regression coefficient	95 % CI	*P* value	Regression coefficient	95 % CI	*P* value
Sex after childbirth	0.64	0.55 to 0.72	<0.0001	0.83	0.74 to 0.93	<0.0001
Faster recovery	0.24	0.18 to 0.30	<0.0001	0.18	0.11 to 0.25	<0.0001
Less painful	0.18	0.12 to 0.24	<0.0001	0.36	0.29 to 0.43	<0.0001
Avoid Episiotomy	0.11	0.02 to 0.19	0.0146	0.34	0.24 to 0.43	<0.0001
Possibility of scheduling delivery	−0.02	−0.11 to 0.07	0.6314	0.17	0.08 to 0.27	0.0004

The analysis showed that women were consistent in their responses (90 % in the private sector and 83 % in the public sector). The consistency was 87 % at the private sector and 79 % at the public sector (please see Additional file 1).

### Follow-up at delivery

**Fig. 2:**
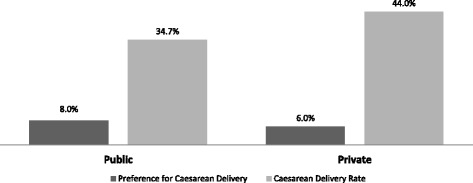
Preference for Cesarean Section and Cesarean Section Rate by healthcare sector

The rates of CS were 43.7 % in the private sector (80/183) and 34.7 % in the public sector (69/199), showing no statistically significant difference (*p* = 0.06) (Fig. 2). Among women in the private sector who expressed a preference for vaginal delivery (*n* = 160), 40 % had a cesarean; 11.3 % (18/160) were elective and 28.9 % (46/160) were intrapartum. In the public sector 33.9 % (62/183) of women that preferred a vaginal delivery had a CS; 4.9 % were elective (9/183) and 27.9 % were intrapartum (51/183). Among women whose preference was for a CS, 43.7 % (7/16) and 72.7 % (8/11) had a cesarean in private and public hospitals, respectively. The rate of instrumental deliveries was 3.3 % in the private sector and 1.0 % in the public sector.

The most frequent indication for cesarean section was labor arrest disorders (arrest of dilatation and/or descent) in both public and private sectors (52.2 and 45.0 %, respectively).

## Discussion

We observed that the majority of women preferred to deliver vaginally. Only 8 % of women in the public sector and 6 % in the private sector stated a preference for cesarean section. Fear of pain and safety were the most frequent expressed reasons for preferring cesarean section, whereas women who preferred vaginal delivery felt it was the most natural mode. However, when women had to evaluate which were the most important attributes of their preferred mode of delivery among a pre-defined list of factors, the quality of sex after childbirth exhibited the strongest association, followed by a fast recovery, less painful experience, no episiotomy, and the possibility of scheduling the delivery, in order of decreasing strength. Finally, women who expressed a preference for vaginal delivery had 34 and 40 % CS rates in public and private hospitals, respectively.

This study has several strengths: a) the high rate of women’s follow-up until delivery is an advantage, as only 1 woman of 382 was lost to follow-up; b) the use of a novel methodology to assess women’s preferences: the DCE; c) the contribution of formative research prior to the study to adapt the questionnaire and to define DCE attributes; and d) the questionnaire and the DCE were piloted prior to this study in similar settings.

There were also some limitations to this study. The selection of hospitals was a convenience sampling and could raise concerns about the representativeness of the sample. That we used a consecutive, non-random sampling technique to select women, and the relatively small overall sample size, and the low preference for CS in our sample, may limit the generalizability of our conclusions.

Only a small proportion of women stated a preference for cesarean section, with marginal differences between women in the public (8 %) versus the private sector (6 %). These rates are similar to or lower than those reported in other surveys that have been conducted during pregnancy in comparable samples of women in Brazil and Chile between 1998 and 2003. Potter and colleagues reported CS preference rates of 10 and 16 % in women attended in the public and private sector respectively, while Angeja and colleagues reported 11 and 8 % CS preference rates, also respectively [9, 11]. Furthermore, comparable surveys in high-income countries between 1999 and 2005 found preference rates between 3 and 17 %, [7, 8, 12, 13, 26]. Thus, in light of the current evidence, our findings suggest that, to date, there is no evidence of either secular changes in women’s preferences for mode of delivery, or clear differences in the opinions of women receiving care in different health sectors who likely come from varying socioeconomic backgrounds.

The relative importance of women’s reasons for their preferences differ according to the methods used to assess them. The most noticeable difference is with regard to the relative importance of sex after childbirth. Women did not mention sex after childbirth when asked to provide reasons for preferring VD or CS with open-ended questions, and chose this reason infrequently in close-ended question. These results are consistent with studies that use questionnaires, in which sex after childbirth is mentioned in a few ones, but not ranked as highly important [8, 11–14, 26]. However, the DCE experiment showed that quality of sex after childbirth was the most important attribute among the five studied. As Hundley and colleagues [22] stated, it could be that the DCE allowed women to give a more honest response, and that women may believe that it is not socially acceptable to say that sex after childbirth is very important for them. This possibility is illuminated by the results of the DCE.

Despite the low preference rates for cesarean section and the absence of pre-labor risk factors, more than 1/3 of the participating women had a cesarean section. Moreover, among women who expressed preference for vaginal delivery, the CS rates were 34 and 40 % in public and private hospitals, respectively. Elective CS was the main difference in the rates between sectors, being 6 percentage points higher in private hospitals. These findings are consistent with two studies by Potter and colleagues [9, 31]. A study published by Potter et al. in 2008 showed a high preference for vaginal delivery in both private and public sectors in Brazil: 72.3 and 79.6 % respectively, being the cesarean delivery rate 72 % in the private sector and 31 % in the public sector - 64.4 % had a scheduled cesarean delivery in the private sector compared with 23.7 % in the public sector. The incidence of real medical reasons for a scheduled cesarean section diagnosed before the onset of labor among private sector patients who had no previous cesarean birth and who wanted a vaginal delivery was only 13 %. It appears evident that determinants other than women’s preferences are more important deciding factors related to cesarean section. The different elective CS rates between sectors for women with the same preferences and low-risk profiles also suggest that differences in the organization of prenatal and delivery care across public and private sector facilities influences cesarean rates.

## Conclusions

In conclusion, and consistently with other studies, the preference for cesarean section is low among healthy nulliparous women in Buenos Aires, and is similar to other sites in Latin America and stable over time. The reasons why young, healthy, nulliparous women with no complications during pregnancy, had a rate of more than 35 % cesarean sections are unlikely related to their preferences for mode of delivery. Our research suggests that women are not responsible for the increase in cesarean section rates, as has been anecdotally hypothesized. Research on other factors related to maternal health care, such as health providers and the organization of prenatal and delivery care is needed to prevent unnecessary cesarean sections. Finally, innovative methods are also needed to assist in researching and elucidating the real, and perhaps undisclosed, reasons related to women’s preferences for mode of delivery.

## Additional file

Additional file 1:
**Step-by-step DCE.** (DOCX 176 kb)
