# Supplementary material for: Women’s preferences and mode of delivery in public and private hospitals: a prospective cohort study
Source: BMC Pregnancy Childbirth. 2016 Feb 8;16:34. doi: 10.1186/s12884-016-0824-0 (PMC4746891; doi:10.1186/s12884-016-0824-0)
Supplement: Additional file 1: — Step-by-step DCE. (DOCX 176 kb) [file 12884_2016_824_MOESM1_ESM.docx]

**STEP-BY-STEP DCE**

**Step 1: Identifying the attributes**

The attributes were identified and selected based on the following: literature review, and previous formative research. We have included five attributes that were found important to pregnant women for deciding mode of delivery in the formative research and literature review [15].. We decided to select the following 5 attributes:

a) Possibility of scheduling the date of delivery

b) Episiotomy

c) Sexual function at 6 months post partum

d) Pain during delivery

e) Recovery after delivery

**Step 2: Assigning levels to the attributes**

Each attribute has different levels. The type and number of levels depends on the characteristic of the attribute. For example, for the attribute “Pain during delivery”, the assigned levels were ordinal (“severe pain” is worse than “moderate pain”), and for “Episiotomy” the levels were categorical (Yes/No). It is important that the levels are plausible to allow women to take the exercise seriously.

The selection of levels was based on the results from literature review and the formative research. Levels of the attributes should be realistic and capable to ensure plausibility and seriousness of the scenarios presented to women.

**Step 3: Producing scenarios**

The combination of attributes and levels generated 72 profiles (three attributes with two options plus two attributes with three options = 32 x 23, full factorial design), which were paired with mirror image technique to created 36 choice sets. Thus, one profile is compared with another which levels are the opposite, for example: the profile 00111 is compared with the profile 11022, where each digit indicated the level of the attribute. Moreover, it is known that participants are able to manage between 8 and 16 comparisons. The 36 choice sets were randomly allocated into 3 groups generating three versions of questionnaire with twelve choice sets and two additional choice sets to assess rationality and consistency. The rationality was evaluated using one choice set where one profile was better than the other on all attributes (“ideal profile”). This choice set allowed evaluating if women were making rational choices. The internal consistency on women’s responses was evaluated using one choice set presented twice in the sequence of choice sets.

**Step 4: Administering the questionnaire**

Before starting the exercise the interviewer explained the woman that she would have to choose between two different types of delivery, each type will have different characteristics. Each attribute was explained using separate cards. For example:

After this explanation the woman started the exercise. We sequentially presented to women 14 choice sets in separate cards. Each choice set was composed by two profiles: A and B; each profile describing a combination of different levels of the 5 selected attributes. An example of one choice set is shown in the table below. We asked women to choose which profile they preferred. Twelve of these choice sets were created based on an orthogonal design (using SPSS system).

**Example of DCE choice set card**

|  | **A** | **B** |
| --- | --- | --- |
| **Possibility of schedule the date of delivery** | Yes | No |
| **Episiotomy** | No | Yes |
| **Sexual function at 6 months post partum** | Worst than before delivery | The same than before delivery |
| **Pain during delivery** | Severe | Mild |
| **Recovery after delivery** | Less than 1 week | Between 1 and 2 weeks |
| ***WHAT OPTION DO YOU PREFER? (CHECK WITH AN X)*** |  |  |
